# Feasibility of a wrist-worn wearable device for estimating mental health status in patients with mental illness

**DOI:** 10.3389/fpsyt.2023.1189765

**Published:** 2023-07-20

**Authors:** Kazuyuki Nakagome, Manabu Makinodan, Mitsuhiro Uratani, Masaki Kato, Norio Ozaki, Seiko Miyata, Kunihiro Iwamoto, Naoki Hashimoto, Atsuhito Toyomaki, Kazuo Mishima, Masaya Ogasawara, Masahiro Takeshima, Kazumichi Minato, Toshikazu Fukami, Mari Oba, Kazuyoshi Takeda, Hideki Oi

**Affiliations:** ^1^Department of Psychiatry, National Center of Neurology and Psychiatry, Kodaira, Japan; ^2^Department of Psychiatry, Nara Medical University, Kashihara, Japan; ^3^Department of Neuropsychiatry, Kansai Medical University, Hirakata, Japan; ^4^Pathophysiology of Mental Disorders, Nagoya University Graduate School of Medicine, Nagoya, Japan; ^5^Department of Psychiatry, Nagoya University Graduate School of Medicine, Nagoya, Japan; ^6^Department of Psychiatry, Hokkaido University Graduate School of Medicine, Sapporo, Japan; ^7^Department of Neuropsychiatry, Akita University Graduate School of Medicine, Akita, Japan; ^8^TechDoctor Inc., Tokyo, Japan; ^9^Department of Clinical Data Science, National Center of Neurology and Psychiatry, Kodaira, Japan

**Keywords:** anxiety, heart rate, Mental Illness Registry, quality of life, wearable device

## Abstract

**Object:**

Real-world data from wearable devices has the potential to understand mental health status in everyday life. We aimed to investigate the feasibility of estimating mental health status using a wrist-worn wearable device (Fitbit Sense) that measures movement using a 3D accelerometer and optical pulse photoplethysmography (PPG).

**Methods:**

Participants were 110 patients with mental illnesses from different diagnostic groups. The study was undertaken between 1 October 2020 and 31 March 2021. Participants wore a Fitbit Sense on their wrist and also completed the State–Trait Anxiety Inventory (STAI), Positive and Negative Affect Schedule (PANAS), and EuroQol 5 dimensions 5-level (EQ-5D-5L) during the study period. To determine heart rate (HR) variability (HRV), we calculated the sdnn (standard deviation of the normal-to-normal interval), coefficient of variation of R-R intervals, and mean HR separately for each sleep stage and the daytime. The association between mental health status and HR and HRV was analyzed.

**Results:**

The following significant correlations were found in the wake after sleep onset stage within 3 days of mental health status assessment: sdnn, HR and STAI scores, HR and PANAS scores, HR and EQ-5D-5L scores. The association between mental health status and HR and HRV was stronger the closer the temporal distance between mental health status assessment and HR measurement.

**Conclusion:**

A wrist-worn wearable device that measures PPG signals was feasible for use with patients with mental illness. Resting state HR and HRV could be used as an objective assessment of mental health status within a few days of measurement.

## 1. Introduction

In the field of psychiatry, the validity of clinical diagnoses is low (1). Because specific diagnostic categories encompass a wide variety of pathologies, elucidation of the etiology and pathophysiology of psychiatric disorders based on existing diagnostic criteria is fraught with difficulty. For this reason, we developed the Mental Illness Registry, in which data items were selected with an awareness of the need to move beyond diagnostic categories to a research approach that focuses on functional domains based on specific neural circuits (the research domain criteria framework) (2). However, to elucidate the biological pathogenesis of functional domains across a wide range of diagnostic categories, it is necessary to establish a research infrastructure with a large patient registry. To obtain a sufficient sample size, patient information was divided into three layers, taking into consideration feasibility based on the function of the medical institution from which patients were recruited. First layer information, which comprised basic clinical information such as demographics, was collected as extensively as possible from all types of psychiatric facilities. Second layer information, which includes clinical evaluation reflecting functional domains, and third layer information, which consists of biometric samples (blood, spinal fluid, brain neuroimaging, genome, and induced pluripotent stem cells) were collected at a medical institution where possible. Electronic patient-reported outcome follow-up using a smartphone was conducted longitudinally to directly access patients and enter self-reported information on anxiety, mood, sleep, social functioning, subjective quality of life (QOL), medications, and other measures.

However, because of concerns about the time and effort involved in self-administered questionnaires, an objective and less burdensome method of monitoring mental health status using wearable devices has attracted attention. One way of estimating mental health status is to use heart rate (HR) variability (HRV), as HR is controlled by the autonomic nervous system (ANS) (3). The parasympathetic nervous system slows HR via the vagus nerve, whereas the sympathetic nervous system accelerates HR through the activation of β-adrenergic receptors. In addition, low HRV is associated with sympathetic nervous system activation that promotes states such as fear and excitement, and higher HRV is associated with parasympathetic nervous system activation, which promotes a state of rest and recovery (3). Lower HRV has been associated with fatigue and pain symptoms of chronic fatigue syndrome, myalgic encephalomyelitis, and fibromyalgia, as well as chronic mental health disorders like anxiety and stress (4–8).

There is growing interest in the use of wrist-worn wearable devices that can measure movement using a 3D accelerometer and optical pulse photoplethysmography (PPG) to obtain biometric information in the real world, rather than in a special environment such as a laboratory. However, the validity of such data for the assessment of patients with mental illness has not been adequately demonstrated. HRV measures the variation in time between heartbeats and is usually calculated from electrocardiogram (ECG) R-R intervals; however, wearable devices record an optical PPG signal and therefore the peak of the R waves cannot be detected accurately. The accuracy of PPG-based HR data has been previously reviewed and validated in comparison with ECG data; PPG-based HR data is typically accurate within a 10% error range (9). As research suggests that wrist-worn devices provide a reliable measure of HRV while the user is in a resting state with no sources of interference, we attempted to test the use of such a device in different phases of the sleep–wake cycle.

In this study, we examined the feasibility and validity of using wearable devices to estimate mental health status in patients enrolled in the Mental Illness Registry, using standardized tests for anxiety, affect, and QOL as a reference. As a wearable device, Fitbit Sense was chosen because Fitbit device is the most researched (9), widely available, relatively inexpensive, and likely to lead to social adoption. The sensor of the PPG is an optical sensor with a green LED, but there are no official statements on other various features such as sensor calibration. We specifically aimed to explore the relationship between scores obtained from self-administered mental health status questionnaires and HR and HRV indices obtained from a wrist-worn wearable device (Fitbit Sense) that measures movement using a 3D accelerometer and optical pulse PPG separately for each sleep stage and the daytime. We reasoned that if the data supported such a relationship, PPG data could be used (at least partly) to assess mental health status instead of self-administered questionnaires, thereby reducing the burden on patients.

## 2. Methods

### 2.1. Patients

A total of 110 patients who were enrolled in the Mental Illness Registry during the study period between 1 October 2020 and 31 March 2021 and provided their informed consent, participated in the study. Participants were either inpatients or outpatients at six sites: the National Center of Neurology and Psychiatry Hospital, Nara Medical University Hospital, Kansai Medical University Hospital, Nagoya University Hospital, Hokkaido University Hospital, and Akita University Hospital. The sample included patients with a wide range of clinical presentations from different diagnostic groups (Table 1). Most patients had a long illness and were in a chronic stage; however, the illness duration of patients with developmental disorders is not shown owing to difficulty determining onset age. At the time of consent, two-thirds of the patients were outpatients, and the rest were inpatients. The daily dose level of antipsychotics (chlorpromazine-equivalent dose), antidepressants (imipramine-equivalent dose), and anxiolytics and hypnotics (diazepam-equivalent dose) was calculated using the conversion table of Inada and Inagaki (10). As expected, antipsychotic dose level was higher in patients with schizophrenia and schizoaffective disorder, and antidepressant dose level was higher in patients with depression. Benzodiazepine dose level was higher in patients with other disorders (“Others” category in Table 1); the State–Trait Anxiety Inventory (STAI) scores of these patients indicated that they were highly anxious and tended to be dependent (as can be inferred from the presence in this category of patients with alcohol-related disorders).

**Table 1:** Patient demographics and clinical characteristics.

Characteristics, mean (SD)	Overall *n* = 110	Schizophrenia or schizoaffective disorder *n* = 25	Bipolar disorder *n* = 15	Depression *n* = 23	Sleep disorder^†^ *n* = 18	Developmental disorder^‡^ *n* = 9	Others^§^ *n* = 20
Age, years	41.7 (14.1)	38.6 (12.2)	43.5 (10.7)	48.6 (16.5)	43.1 (13.6)	38.9 (11.6)	36.7 (15.1)
Sex, male (%)	40 (36.4%)	9 (36.0%)	7 (46.7%)	9 (39.1%)	10 (55.6%)	3 (33.3%)	2 (10.0%)
Duration, years	10.5 (9.1)	14.5 (11.8)	14.2 (6.7)	8.2 (5.9)	4.8 (4.4)		10.7 (11.2)
Setting, inpatient (%)	39 (35.5%)	16 (64.0%)	2 (13.3%)	8 (34.8%)	3 (16.7%)	1 (11.1%)	9 (45.0%)
BMI^¶^	23.9 (5.8)	26.3 (7.7)	25.9 (4.0)	22.1 (3.6)	25.1 (4.3)	26.3 (6.8)	20.4 (5.7)
Mental health status							
STAI-1	47.3 (12.0)	41.1 (9.5)	44 (8.5)	51.3 (13.5)	45.9 (8.1)	48 (14.6)	53.5 (13.1)
STAI-2	53.9 (11.2)	48.9 (6.1)	50.9 (6.8)	54.2 (15.1)	54.7 (10.1)	57.1 (9.5)	59.6 (12.5)
PANAS, PA	21.2 (6.9)	23.1 (5.9)	22 (5.2)	21.3 (8.3)	18 (6.3)	21.2 (7.7)	21.1 (7.2)
PANAS, NA	22.7 (8.5)	19.9 (6.3)	20.9 (6.6)	23.7 (10.0)	19.3 (7.6)	27.8 (7.5)	26.8 (9.4)
EQ-5D-5L, VAS	62.8 (22.9)	68.5 (20.7)	70.9 (15.6)	52.5 (25.5)	67.7 (20.5)	59.8 (24.5)	58.7 (25.4)
EQ-5D-5L, utility score	0.7 (0.3)	0.8 (0.3)	0.8 (0.2)	0.5 (0.3)	0.7 (0.3)	0.6 (0.2)	0.7 (0.3)
Medication (daily dose level)							
CP equiv.	180.7 (310.3)	484.9 (413.4)	179.6 (316.7)	56.6 (182.6)	55.3 (146)	78.2 (149.6)	103 (170.2)
IMI equiv.	54.6 (91.2)	19.9 (44.7)	7.5 (21.0)	123.9 (124.4)	37.4 (91.6)	91.7 (105.3)	52.5 (68.3)
DZ equiv.	5.8 (8.5)	4.7 (5.0)	1.4 (2.9)	7.1 (8.1)	4.1 (8.8)	4.4 (8.5)	11.4 (12.0)

SD, standard deviation; BMI, body mass index; STAI-1, State–Trait Anxiety Inventory Form X-I; STAI-2, State–Trait Anxiety Inventory Form X-II; PANAS, the Positive and Negative Affect Schedule; PA, positive affect; NA, negative affect; EQ-5D-5L, EuroQol 5 dimensions 5-level; VAS, visual analog scale; CP equiv., chlorpromazine-equivalent dose; IMI equiv., imipramine-equivalent dose; DZ equiv., diazepam-equivalent dose.

† Insomnia (*n* = 6), sleep apnea, sleep–wake rhythm disorder (*n* = 4), hypersomnia, restless legs syndrome, REM sleep behavior disorder, sleep-related eating disorder (*n* = 1).

‡ Autism spectrum disorder (*n* = 6), attention-deficit/hyperactivity disorder (*n* = 3).

§ Anorexia nervosa (*n* = 4), dissociative disorder, alcohol-related disorder (*n* = 3), obsessive–compulsive disorder, panic disorder (*n* = 2), epilepsy, symptomatic psychosis, somatoform disorder, social anxiety disorder, borderline personality disorder, personality disorder not otherwise specified (*n* = 1).

¶ There are missing values for BMI. Overall (*n* = 74), schizophrenia or schizoaffective disorder (*n* = 16), bipolar disorder (*n* = 7), depression (*n* = 17), sleep disorder (*n* = 15), developmental disorder (*n* = 5), others (*n* = 14).

The Ethics Committee of the National Center of Neurology and Psychiatry approved the study protocol and experimental procedures (B2021-086).

### 2.2. Procedures

This was a multisite observational study. Participants were either inpatients or outpatients at six sites: the National Center of Neurology and Psychiatry Hospital, Nara Medical University Hospital, Kansai Medical University Hospital, Nagoya University Hospital, Hokkaido University Hospital, and Akita University Hospital. The software setup for data acquisition was conducted in advance by the researchers. Participants had only to wear a Fitbit Sense on their left or right wrist for as long as they could tolerate it between the day of consent and their next visit to the hospital (for outpatients), or until their first visit to the hospital after discharge (for inpatients). While wearing the Fitbit Sense, participants were contacted via their smartphones to inquire about their current socioenvironmental situation and to ask them to complete the following self-administered questionnaires: the STAI FORM X-I, II (11) (to measure anxiety), the Positive and Negative Affect Schedule (PANAS) (12) (to measure affect), and the EuroQol 5 dimensions 5-level (EQ-5D-5L) (to measure QOL).

### 2.3. HRV measurement and sleep stage estimation

To measure HRV, we used time-domain indices such as sdnn (standard deviation of the normal-to-normal interval) and cvrr (coefficient of variation of R-R intervals), which are modulated by the ANS. We also assessed HR. The sdnn, cvrr, and HR were calculated using a sampling time of 5 s with 30 min as one epoch; if there were multiple epochs appropriately recorded, the average value was used as the representative value. Assuming that during the daytime HR is sensitive to activity, we excluded data in which the HR was greater than 90/min or fell below 50/min, and in which the difference between the maximum and minimum within one epoch was greater than 40/min or less than 5/min. During sleep, only the latter condition was used; data in which the difference between the maximum and minimum HRs within one epoch was greater than 40/min or less than 5/min were excluded because of the suspected possibility of measurement noise. The Fitbit device used in the present study records an optical PPG signal and thus the peak of the R waves cannot be detected accurately. Therefore, the interval between the pulse wave (estimated by 60/mean HR) was used as a surrogate for the R-R intervals obtained from an ECG.

Sleep stage information in this study was provided by the Fitbit Research Library, which analyzed the 3D accelerometer and optical PPG data collected in this study. Unfortunately, the algorithm for determining sleep stage from 3D accelerometer and PPG data obtained from Fitbit is not publicly available. However, Beattie et al. provides an overview of the analysis using the generated features on a 30 s time scale based on motion, HRV, and breathing rate parameters were used to develop an automated sleep staging algorithm (13). Specifically, an initial set of 180 features was calculated for each 30 s epoch and fed into an automated classifier, together with the gold standard labels provided by polysomnography. This classifier outputs labels of “wake after sleep onset (WASO),” “light,” “deep,” or “REM” for each 30 s epoch; the gold standard labels are used to choose the optimal set of classifier parameters. Instead of expecting to provide a precise estimation of sleep stage, it was aimed to identify the correlations between true sleep stages and physiological indices such as movement and HRV, and to develop a machine learning algorithm that uses these correlations to approximate the most likely underlying sleep stages. The overall per-epoch accuracy of the algorithm was 69%, with a Cohen’s kappa of 0.52, which indicates a reasonable level of sleep staging accuracy in health populations (13). We also evaluated the accuracy of Fitbit sleep stage determination in another study of patients enrolled in the Mental Illness Registry, using PSG as a criterion. The results showed that the Fitbit Sense had an overall per-epoch accuracy for each sleep stage in the range of 0.61–0.84, which seemed fair (14). HR and HRV indices were calculated and submitted for analysis if the HR was recorded continuously and appropriately for 30 min epoch within each sleep stage.

### 2.4. Mental health status assessment

As reference, we used electronic self-reported assessments of anxiety, affect, and QOL. Patients reported their data using a smartphone. The following self-administered questionnaires were used: the STAI Form X-I and Form X-II to measure state and trait anxiety, the PANAS to measure positive and negative affect, and the EQ-5D-5L visual analog scale (VAS) and utility score to measure QOL.

The STAI Form X-I and Form X-II were developed by Spielberger et al. (15); they have been translated into Japanese and validated using 618 college students (11). The STAI Form X-I is used to assess anxiety as a situation-related state, and the STAI Form X-II assesses anxiety as a relatively stable personality trait. Each questionnaire subscale consists of 20 items (including reverse items) that comprise short statements about an individual’s subjective feelings. Respondents are asked to select one of four response choices for each item: 1 = not at all/almost never, 2 = somewhat/sometimes, 3 = moderately so/often, and 4 = very much so/almost always. The minimum score for each scale is 20, with a maximum of 80; higher scores indicate greater anxiety.

The PANAS consists of 20 items that comprise adjectives representing positive and negative affect (16). The reliability and validity of the Japanese version of the PANAS have been demonstrated in 1290 healthy volunteers using a 6-point Likert response scale ranging from 1 = not at all to 6 = very much so (12). Respondents are asked to select one of four response choices. The 10 positive affect items are interested, excited, strong, enthusiastic, proud, alert, inspired, determined, attentive, and active. The 10 negative affect items are distressed, upset, guilty, scared, hostile, irritable, ashamed, nervous, jittery, and afraid. Positive affect and negative affect scores are calculated separately.

The EQ-5D is a preference-based measure of QOL based on general health dimensions and has been used widely in both clinical trials and health services research (17, 18). This measure is based on the expected utility theory and provides a single measure (the “utility score”) that captures the respondent’s QOL from multiple perspectives. The EQ-5D-5L consists of two parts. One part comprises a classification system of five dimensions (5D; mobility, self-care, usual activities, pain/discomfort, and anxiety/depression) with five levels of response options (5 L; no problems, slight problems, moderate problems, severe problems, and unable to/extreme problems) per dimension. The other part comprises a VAS (18). The 5 L classification system defines 3125 (i.e., 5^5^) possible health states, and utility scores are derived from a preference-based algorithm used to calculate quality-adjusted life years (which are often used in the economic evaluation of medical technology). In this study, an algorithm developed using survey results based on the composite time trade-off in Japan was used (19). The VAS records respondents’ current self-rated general health on a line ranging from 0 (the worst imaginable health) to 100 (the best imaginable health).

### 2.5. Statistical analysis

To investigate the relationship between HR and HRV indices and self-reported mental health status, participants who did not have at least one HR and HRV epoch within 2 weeks of the mental health status assessment were excluded from the analysis. First, exploratory univariate correlation analyses (Pearson’s product moment correlation) were performed to examine associations between HR and HRV indices (sdnn, cvrr) and mental health status variables (STAI Form X-I and Form X-II scores, PANAS positive affect and negative affect scores, and EQ-5D-5L VAS and utility scores) for each sleep stage and the daytime to estimate which time period HR and HRV indices best reflected mental health status. Then, to explore the effect of focusing on the period showing the strongest association, the effect of temporal distance between the time of HR measurement and mental health status assessment on the association between them was examined using univariate correlation analyses (Pearson’s product moment correlation) between HR and HRV indices and mental health status variables when HR and HRV data were restricted to within 7 days and within 3 days of the mental health status assessment. For the data set that was ultimately estimated to have the strongest association, multivariable regression analysis was performed with each HRV index, age, sex, chlorpromazine-equivalent of antipsychotics, imipramine-equivalent dose of antidepressants, and diazepam-equivalent dose of anxiolytics and hypnotics as independent variables, and each mental health status variable as a dependent variable, to adjust for possible confounding factors (3, 20, 21).

The statistical analysis was performed using JMP version 13.0.0 (SAS Institute Inc., Cary, NC, USA); a *P*-value of <0.05 was considered statistically significant for all tests.

## 3. Results

**Figure 1:**
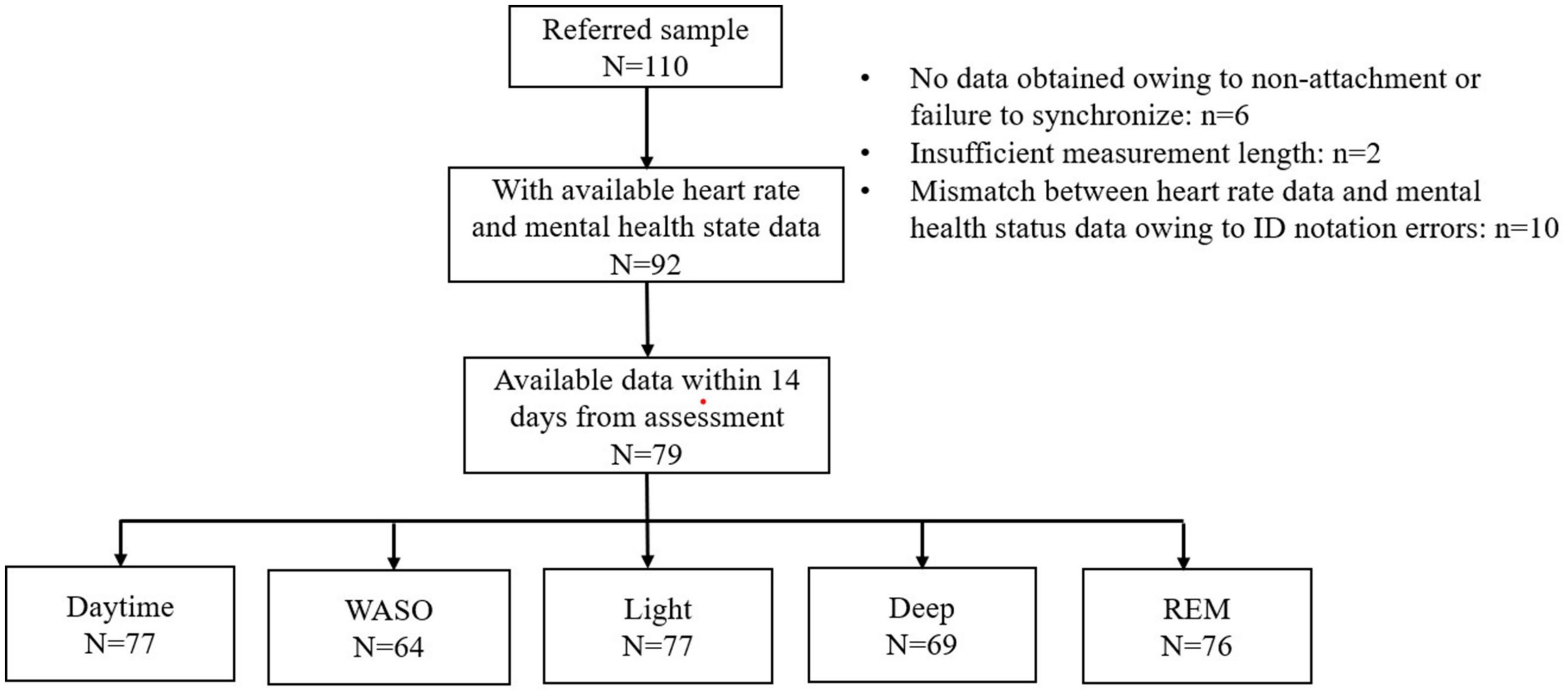
Flow diagram. A total of 110 patients were referred to the study. Of these, 92 had at least one epoch (30 min) of available heart rate data with mental health status data. A total of 13 of the 92 patents were excluded because the heart rate data was not obtained within 14 days of mental health status assessment. The figure shows the number of patients from whom available heart rate data were obtained for each sleep stage and the daytime. WASO, wake after sleep onset.

Of the 110 patients who consented to participate in the study, available data on HRV indices were obtained from 92 patients (Figure 1). For these 92 patients, the mean number of measurement days was 8.4 days, ranging from 1 to 38 days (Supplementary Figure 1A). The HR and HRV data were mostly obtained within 14 days from the mental health status assessment date (Supplementary Figure 1B). In fact, 79 patients had at least one epoch for HRV indices within 14 days (Figure 1). Figure 1 shows the numbers of patients who had available data for each sleep stage and the daytime.

**Figure 2:**
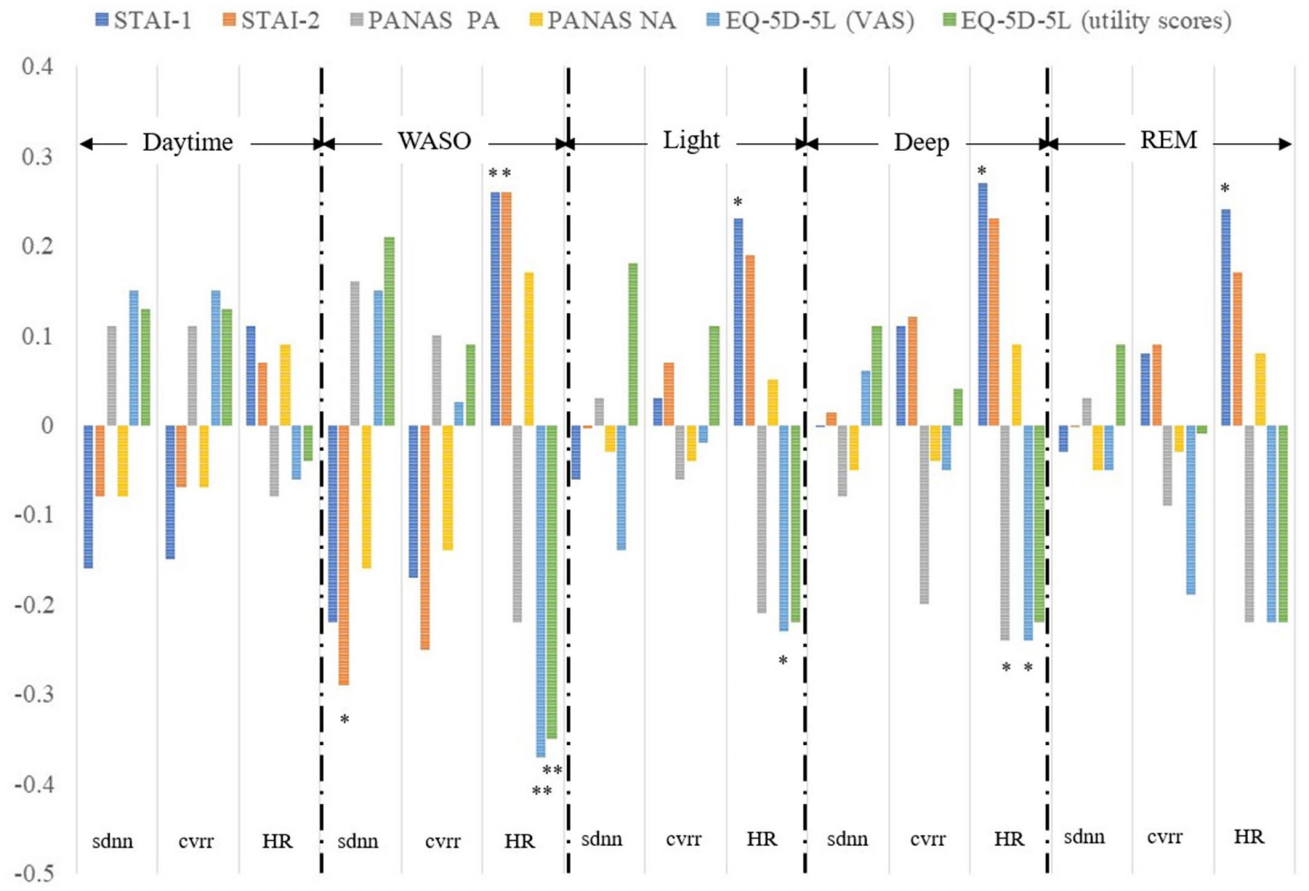
Correlation between HRV and HR indices and mental health status variables. Correlations between sdnn, cvrr, and HR and scores for the STAI-1 and 2, PANAS PA and NA, EQ-5D-5L VAS and utility obtained within 14 days were calculated separately for each sleep stage and the daytime. sdnn, standard deviation of the normal-to-normal interval; cvrr, coefficient of variation of R-R interval; HR, mean heart rate; STAI-1, State–Trait Anxiety Inventory Form X-I; STAI-2, State–Trait Anxiety Inventory Form X-II; PANAS, the Positive and Negative Affect Schedule; PA, positive affect; NA, negative affect; EQ-5D-5L, EuroQol 5 dimensions 5-level; VAS, visual analog scale; WASO, wake after sleep onset. **P < 0.01, *P < 0.05.

The exploratory analyses of correlations between HR and HRV indices and mental health status variables for each sleep stage and the daytime showed a significant correlation between sdnn and STAI Form X-II scores, between HR and STAI Form X-I and Form-II scores, between HR and EQ-5D-5L VAS and utility scores in the WASO stage; between HR and STAI Form X-I scores and EQ-5D-5L VAS scores in the light stage; between HR and STAI Form X-I scores, PANAS positive affect scores, and EQ-5D-5L VAS scores in the deep stage; and between HR and STAI Form X-I scores in the REM stage (Figure 2). As can be seen from Figure 2, the association between HRV indices and mental health status variables was strongest in the WASO stage.

Focusing on data obtained in the WASO stage, the correlations between HR and HRV indices and mental health status variables within 14 days, 7 days, and 3 days of the time of mental health status assessment are shown in Table 2. As shown in Table 2, the association between HR and HRV indices and mental health status assessment was strongest for data obtained within 3 days from the time of mental health status assessment.

**Table 2:** Pearson correlations between HR and HRV and mental health status variables for different time intervals from mental health status assessment.

					STAI-1		STAI-2		PANAS (PA)		PANAS (NA)		EQ-5D-5L (VAS)		EQ-5D-5L (utility scores)	
sdnn		*n*	Measurement length (min)^†^	Center of gravity (day)^‡^	*r*	*P*	*r*	*P*	*r*	*P*	*r*	*P*	*r*	*P*	*r*	*P*
	±3 days	41	69.3 ± 49.36	1.8 ± 0.82	−0.33*	0.036	−0.48**	0.0017	0.22	0.15	−0.25	0.11	0.23	0.14	0.23	0.16
	±7 days	55	103.7 ± 78.78	3.5 ± 1.46	−0.19	0.16	−0.33*	0.014	0.17	0.22	−0.16	0.25	0.13	0.33	0.16	0.24
	±14 days	63	107.3 ± 87.47	4.4 ± 2.81	−0.22	0.09	−0.29*	0.021	0.16	0.21	−0.16	0.21	0.15	0.24	0.21	0.10
cvrr																
	±3 days	41	69.3 ± 49.36	1.8 ± 0.82	−0.24	0.13	−0.43**	0.0052	0.10	0.52	−0.24	0.13	0.08	0.64	0.07	0.65
	±7 days	55	103.7 ± 78.78	3.5 ± 1.46	−0.16	0.25	−0.31*	0.022	0.09	0.49	−0.17	0.20	0.02	0.89	0.04	0.75
	±14 days	63	107.3 ± 87.47	4.4 ± 2.81	−0.17	0.2	−0.25	0.051	0.10	0.44	−0.14	0.26	0.03	0.85	0.09	0.48
Heart rate																
	±3 days	41	69.3 ± 49.36	1.8 ± 0.82	0.44**	0.0043	0.35*	0.026	−0.43**	0.0045	0.17	0.27	−0.53***	0.0004	−0.50***	0.0008
	±7 days	55	103.7 ± 78.78	3.5 ± 1.46	0.22	0.11	0.22	0.11	−0.26	0.05	0.07	0.6	−0.34*	0.01	−0.34*	0.012
	±14 days	63	107.3 ± 87.47	4.4 ± 2.81	0.26*	0.041	0.26*	0.043	−0.22	0.077	0.17	0.18	−0.37**	0.0028	−0.35**	0.0047

sdnn, standard deviation of the normal-to-normal interval; cvrr, coefficient of variation of R-R interval; STAI-1, State–Trait Anxiety Inventory Form X-I; STAI-2, State–Trait Anxiety Inventory Form X-II; PANAS, the Positive and Negative Affect Schedule; PA, positive affect; NA, negative affect; EQ-5D-5L, EuroQol 5 dimensions 5-level; VAS, visual analog scale.

† The average length used for measuring the heart rate within the time range.

‡ Center of gravity = Sum (measurement length (for each day) * distance (days) from the mental health status assessment)/(total measurement length).

*P < 0.05; **P < 0.01; ***P < 0.001.

Multivariable regression analyses were conducted on the variables that showed significant intercorrelations for data obtained within 3 days of mental health status assessment (Supplementary Table 1). After adjusting for possible confounders, the following significant associations were demonstrated: sdnn and STAI Form X-II scores (Est = −0.14, 95% CI [−0.26, −0.01], *P* = 0.035); HR and STAI Form X-I scores (Est = 0.41, 95% CI [0.03, 0.78], *P* = 0.036); HR and PANAS positive affect scores (Est = −0.36, 95% CI [−0.59, −0.12], *P* = 0.004); HR and EQ-5D-5L VAS scores (Est = −0.98, 95% CI [−1.67, −0.29], *P* = 0.007); and HR and EQ-5D-5L utility scores (Est = −0.01, 95% CI [−0.02, 0.00], *P* = 0.016).

## 4. Discussion

In this study, we explored the feasibility of using PPG obtained by a wrist-worn wearable device to assess mental health status in patients with mental illness. Of 110 patients, 92 (83.6%) returned usable HR data. HR and HRV measured within 14 days of the mental health status assessment were significantly associated with anxiety and QOL in different sleep stages, and the association was strongest in the WASO stage. Although exploratory, the results also suggest that HR and HRV in the WASO stage may reflect anxiety, positive affect, and QOL within 3 days.

ECG data on HR and HRV are one of the most important biomarkers of emotion (22). When an individual is exposed to stress, changes occur in HR, HRV, and other physiological signals regulated by the ANS. As a potential surrogate for ECG measurements, wearable PPG sensors enable continuous monitoring of HR and HRV and thus reflect real-time stress levels and mental health status. It has been observed that PPG measurement levels of HRV decrease as stress increases (23) However, there are insufficient studies on the relationship between PPG measures of HRV and anxiety and depression in clinical settings. Cakmak et al. (24) investigated whether a PPG-based watch could predict post-traumatic stress disorder outcomes (e.g., pain, sleep, and anxiety). Participants were divided into three subgroups according to data collection methods: (1) patients who wore the watch to collect HRV and actigraphy data, (2) patients who answered a survey, and (3) patients who both wore the watch and answered a survey. The highest performance of watch-based features was achieved for classifying participants with pain by a logistic regression model, with an area under the receiver operating characteristic curve (AUC) of 0.70. The survey-based model achieved an AUC of 0.77, and the fusion of watch and survey metrics slightly improved the AUC to 0.79. The accuracy of the wearable data was considered acceptable, indicating that wearable PPG sensors are potentially useful for post-traumatic stress disorder monitoring. Sheridan et al. investigated the relationship between HRV changes and suicidality as measured prospectively by the Columbia Suicide Severity Scale in acutely suicidal adolescents (25). Using frequency domain HRV indices they found an inverse correlation between suicidality and the high frequency component, which reflects parasympathetic activity. Interestingly, 104 acutely suicidal adolescents were enrolled in the study; however, after excluding inappropriate data, only 51 patients remained in the final cohort. Of the 53 excluded participants, 38 were excluded because they had no artifact-free data. This was so even though at least 1 min of artifact-free data were collected every hour, because PPG is susceptible to motion artifact, unlike ECG. In the present study, although participants were asked to wear the Fitbit device for as long as possible, longitudinal monitoring was not performed because the data used for analysis were obtained from several 30-min epochs of continuous, artifact-free data. Wrist-worn devices are reported to be less uncomfortable than devices worn on the chest (26) however, if devices are worn for a prolonged time, it may lead to adverse events such as contact dermatitis. Therefore, we chose to obtain a physiological marker that reflects mental health status obtained by wearing the device for a limited time. The results suggest that HR and HRV data related to mental health status may be obtained with an average measurement time of approximately 1 h during the WASO stage, which is within 3 days of the mental health status assessment (Table 2). Sheridan et al. (27) considered that the conventional short-term measurement standard of 5 min (3) was still too lengthy to obtain stable data from a wrist-worn wearable device. They analyzed the correlation between commonly used HRV indices (time domain and frequency domain) at 1 min and those at 5 min and found that both indices showed greater than moderate correlations. How much the measurement time can be reduced is an issue that may be resolved in the future.

Our findings suggest that HR and HRV in the WASO stage better reflect current mental health status than data in other sleep stages and in the daytime. This may be because the resting state is maintained during the WASO stage, as it is less affected by daytime activities or ANS activity during REM sleep. In addition, the accuracy of wearable devices in estimating sleep stages in healthy individuals has been reported as reasonable (13, 14). One previous study found that a wearable device correctly estimated approximately 70% of participants as being in the WASO stage; approximately 23% of them were actually in the light sleep stage according to polysomnography. Therefore, it is likely that participants estimated as being in the WASO stage in the present study were in either the WASO or light sleep stage. Although HR and HRV data during the WASO stage were most strongly associated with mental health status, the number of participants for whom data were available within 14 days was the lowest in this stage (64 out of 79) compared with the daytime and other sleep stages. This may be because of the short duration of this stage, which made it difficult to obtain stable epochs longer than 30 min; if the epoch time for calculating HRV could be reduced, which Sheridan et al. (27) have suggested is feasible, the number of dropouts could be reduced.

A recent review (28) found that 12 of the 20 studies on stress used a PPG sensor alone to monitor stress; in the remaining 8 studies, a PPG sensor was used in combination with other sensors. In general, a higher performance is achieved if a PPG sensor is used in combination with other sensors than if PPG is used alone (29–31). Indeed, approaches that use machine learning algorithms combining PPG with other physiological indices, such as ECG, electroencephalography, near-infrared spectroscopy, accelerometers/gyroscopes, and galvanic skin response, seem very promising. However, research to date has yet to reach a consensus on the best classifier for detecting stress, indicating that the performance of the classification algorithms may depend on the dataset. Moreover, how many classifications should be made has not been determined. Although the most common classifiers are binary (either stress is detected or not), in the real world, stress is often experienced at various levels as a continuum, suggesting that there are many issues to be resolved regarding appropriate methodologies.

The present study had several limitations. Although a common technique is to develop a peak detector algorithm to detect the peaks in the PPG signal, and the time between PPG peaks is used as a surrogate for the R-R intervals (13) in the present study, HRV was calculated from HR measured with a sample time of 5 s, which may have reduced accuracy. Additionally, we did not confirm the longitudinal changes. In other words, it remains to be confirmed whether HR and HRV indices respond to changes in mental health status within an individual. In particular, although sdnn was significantly associated with the trait-dependent STAI Form X-II scores but not with state-dependent Form X-I scores, it is unclear whether sdnn corresponds to changes in mental health status. Furthermore, several studies have reported that HR, which was most strongly associated with mental health status in the present study, reflects ANS function less accurately than HRV (23, 32). These issues warrant further research, including longitudinal studies. Another study limitation is that we used a specific band-pass filter to exclude unwanted HR and HRV signals following visual inspection; however, there is no solid evidence for the accuracy of this method. Standardization of optimal cutoff ranges is an important issue, although it may be device dependent. Finally, we did not adjust for the multiplicity of analyses. The present results are therefore exploratory and additional studies are needed to confirm them.

This study examined the feasibility and potential of a wrist-worn wearable device to assess the mental health status of mentally ill patients. Although the results were exploratory and cross-sectional, they suggest the potential utility of this method. Additional validation studies, including longitudinal studies, are warranted. If it is possible to objectively monitor mental health status in the home by the wearing of a wrist-worn device for a relatively short period, this could reduce the burden on patients in cohort studies and improve their well-being.

## Data availability statement

The original contributions presented in the study are included in the article/Supplementary material, further inquiries can be directed to the corresponding author.

## Ethics statement

The studies involving human participants were reviewed and approved by the Ethics Committee of the National Center of Neurology and Psychiatry. The patients/participants provided their written informed consent to participate in this study.

## Author contributions

KN contributed toward conceptualization, methodology, investigation, project administration, and writing of the original draft. KMin and TF contributed toward data curation, provision of resources, and formal analysis. MM, MU, MK, NO, SM, KI, NH, AT, MO, MT, and KMis contributed toward conceptualization, methodology, and investigation. MOb, KT, and HO contributed toward conceptualization, data curation, and supervising the statistical analysis. All authors contributed toward acquisition of data, critical revision of the manuscript for important intellectual content, approval of the final version of the manuscript, and agree to be accountable for all aspects of the work and ensure that any questions related to the accuracy or integrity of any part of the work will be appropriately investigated and resolved.

## Funding

This research was supported by AMED under Grant number JP21dk0307103 and an Intramural Research Grant (3-1) for Neurological and Psychiatric Disorders of NCNP. KN confirms that he had full access to all the data in the study and takes final responsibility for the decision to submit for publication.

## Conflict of interest

KN reports grants from AMED during the conduct of this study and from Otsuka Pharmaceutical Co., Ltd., Sumitomo Pharma Co., Ltd. and Janssen Pharmaceutical K.K., and personal fees from Otsuka Pharmaceutical Co., Ltd., Sumitomo Pharma Co., Ltd., Meiji-Seika Pharma Co., Ltd., Janssen Pharmaceutical K.K., MOCHIDA PHARMACEUTICAL CO., LTD., Mitsubishi Tanabe Pharma Corp., Viatris Inc., and Eisai Co., Ltd. He has received payment for advisory work from Takeda Pharmaceutical Co., Ltd., Lundbeck Japan, and Nippon Boehringer Ingelheim Co., Ltd., outside the submitted work. MM has received research grants from Sumitomo Pharma Co., Ltd. and Kyowa Kirin Co., Ltd. MK has received grant funding from the Japanese Ministry of Health, Labour and Welfare, the Japan Society for the Promotion of Science, SENSHIN Medical Research Foundation, the Japan Research Foundation for Clinical Pharmacology, and the Japanese Society of Clinical Neuropsychopharmacology, and speaker honoraria from Sumitomo Pharma Co., Ltd., Otsuka Pharmaceutical Co., Ltd., Meiji-Seika Pharma Co., Ltd., Eli Lilly Japan K.K., Merck & Co., Inc., Pfizer Inc., Janssen Pharmaceutical K.K., Shionogi & Co., Ltd., Mitsubishi Tanabe Pharma Corp., Takeda Pharmaceutical Co., Ltd., Lundbeck Japan, Viatris Inc., Eisai Co., Ltd., and ONO PHARMACEUTICAL CO., LTD., and has participated in advisory/review boards for Otsuka Pharmaceutical Co., Ltd., Sumitomo Pharma Co., Ltd., Shionogi & Co., Ltd., and Nippon Boehringer Ingelheim Co., Ltd. NO has received research support or speaker honoraria from, or has served as a joint researcher with, or a consultant to, Sumitomo Pharma Co., Ltd., Eisai Co., Ltd., Otsuka Pharmaceutical Co., Ltd., KAITEKI, Mitsubishi Tanabe Pharma Corp., Eli Lilly Japan K.K., MOCHIDA PHARMACEUTICAL CO., LTD., DAIICHI SANKYO CO., LTD., TSUMURA & CO., Takeda Pharmaceutical Co., Ltd., Meiji-Seika Pharma Co., Ltd., EA Pharma Co., Ltd., Viatris Inc., Ricoh Co., Ltd., Nippon Boehringer Ingelheim Co., Ltd., Lundbeck Japan, Nihon Medi-Physics Co., Ltd., and Nippon Chemiphar Co., Ltd., outside the submitted work. KI has received speaker honoraria from Eisai Co., Ltd., Kyowa Kirin Co., Ltd., Meiji-Seika Pharma Co., Ltd., Merck & Co., Inc., Otsuka Pharmaceutical Co., Ltd., Sumitomo Pharma Co., Ltd., Taisho Pharmaceutical Co., Ltd., Takeda Pharmaceutical Co., Ltd., TOWA PHARMACEUTICAL CO., LTD., and Viatris Inc., outside the submitted work. NH has received personal fees from Janssen Pharmaceutical K.K., Yoshitomiyakuhin Corporation, Otsuka Pharmaceutical Co., Ltd., Sumitomo Pharma Co., Ltd., Novartis Pharma K.K., Meiji-Seika Pharma Co., Ltd., Lundbeck Japan, and Takeda Pharmaceutical Co., Ltd. AT received a research grant from Nippon Boehringer Ingelheim Co., Ltd. and personal fees from MOCHIDA PHARMACEUTICAL CO., LTD., Sumitomo Pharma Co., Ltd., and Otsuka Pharmaceutical Co., Ltd. KMis has received research grants from Eisai Co., Ltd., Sumitomo Pharma Co., Ltd., Takeda Pharmaceutical Co., Ltd., and Sony Corporation and has also received speaker honoraria from Eisai Co., Ltd., Nobelpharma Co., Ltd., Takeda Pharmaceutical Co., Ltd., Merck & Co., Inc., Otsuka Pharmaceutical Co., Ltd., and Viatris Inc. MT has received speaker honoraria from Takeda Pharmaceutical Co., Ltd., Otsuka Pharmaceutical Co., Ltd., DAIICHI SANKYO CO., LTD., Sumitomo Pharma Co., Ltd., Meiji-Seika Pharma Co., Ltd., Viatris Inc., Merck & Co., Inc., Eisai Co., Ltd., and Yoshitomiyakuhin Corporation outside the submitted work. KMin and TF were employed by TechDoctor Inc.

The remaining authors declare that the research was conducted in the absence of any commercial or financial relationships that could be construed as a potential conflict of interest.

## Publisher’s note

All claims expressed in this article are solely those of the authors and do not necessarily represent those of their affiliated organizations, or those of the publisher, the editors and the reviewers. Any product that may be evaluated in this article, or claim that may be made by its manufacturer, is not guaranteed or endorsed by the publisher.
