# Supplementary material for: Feasibility of a wrist-worn wearable device for estimating mental health status in patients with mental illness
Source: Front Psychiatry. 2023 Jul 20;14:1189765. doi: 10.3389/fpsyt.2023.1189765 (PMC10399687; doi:10.3389/fpsyt.2023.1189765)
Supplement: Supplementary file 1 [file Table_1.DOCX]

| **Supplementary Table 1**. Multivariable regression analyses for variables obtained within 3 days of mental health status assessment and significantly intercorrelated in univariate analyses | | | | | | |
| --- | --- | --- | --- | --- | --- | --- |
|  |  |  |  |  |  |  |
| a. STAI-I | Variables | Coefficient | Standard error | *P* | 95% Confidence interval | |
|  |  |  |  |  | Lower bound | Upper bound |
|  | sdnn | −0.05 | 0.08 | 0.522 | −0.21 | 0.11 |
|  | Sex (M=1, F=2) | 0.84 | 2.05 | 0.686 | −3.32 | 4.99 |
|  | Age | −0.26 | 0.15 | 0.088 | −0.57 | 0.04 |
|  | CP equiv. | −0.01 | 0.01 | 0.119 | −0.02 | 0.00 |
|  | IMI equiv. | 0.03 | 0.02 | 0.190 | −0.01 | 0.07 |
|  | DZ equiv. | 0.41 | 0.23 | 0.081 | −0.05 | 0.88 |
|  |  |  |  |  |  |  |
| b. STAI-Ⅱ | Variables | Coefficient | Standard error | *P* | 95% Confidence interval | |
|  |  |  |  |  | Lower bound | Upper bound |
|  | sdnn* | −0.14 | 0.06 | 0.035 | −0.26 | −0.01 |
|  | Sex (M=1, F=2) | −3.07 | 1.63 | 0.068 | −6.38 | 0.24 |
|  | Age | −0.20 | 0.12 | 0.109 | −0.44 | 0.05 |
|  | CP equiv. | 8e-4 | 5e-3 | 0.873 | −0.01 | 0.01 |
|  | IMI equiv. | 0.01 | 0.02 | 0.385 | −0.02 | 0.05 |
|  | DZ equiv. | 0.08 | 0.18 | 0.657 | −0.29 | 0.45 |
|  |  |  |  |  |  |  |
| c. STAI-Ⅱ | Variables | Coefficient | Standard error | *P* | 95% Confidence interval | |
|  |  |  |  |  | Lower bound | Upper bound |
|  | cvrr | −1.09 | 0.63 | 0.091 | −2.38 | 0.19 |
|  | Sex (M=1, F=2) | −2.99 | 1.67 | 0.082 | −6.39 | 0.40 |
|  | Age | −0.20 | 0.12 | 0.109 | −0.46 | 0.05 |
|  | CP equiv. | 3e-4 | 5e-3 | 0.952 | −0.01 | 0.01 |
|  | IMI equiv. | 0.02 | 0.02 | 0.299 | −0.02 | 0.05 |
|  | DZ equiv. | 0.11 | 0.19 | 0.564 | −0.27 | 0.49 |
|  |  |  |  |  |  |  |
|  |  |  |  |  |  |  |
| d. STAI-Ⅰ | Variables | Coefficient | Standard error | *P* | 95% Confidence interval | |
|  |  |  |  |  | Lower bound | Upper bound |
|  | Heart rate* | 0.41 | 0.19 | 0.036 | 0.03 | 0.78 |
|  | Sex (M=1, F=2) | 0.38 | 1.94 | 0.846 | −3.56 | 4.32 |
|  | Age | −0.27 | 0.14 | 0.052 | −0.55 | 0.00 |
|  | CP equiv. | −0.01 | 0.01 | 0.057 | −0.02 | 0.00 |
|  | IMI equiv. | 0.02 | 0.02 | 0.478 | −0.03 | 0.06 |
|  | DZ equiv. | 0.39 | 0.21 | 0.081 | −0.05 | 0.82 |
|  |  |  |  |  |  |  |
| e. STAI-Ⅱ | Variables | Coefficient | Standard error | *P* | 95% Confidence interval | |
|  |  |  |  |  | Lower bound | Upper bound |
|  | Heart rate | 0.29 | 0.16 | 0.075 | −0.03 | 0.62 |
|  | Sex* (M=1, F=2) | −3.42 | 1.67 | 0.048 | −6.81 | −0.03 |
|  | Age* | −0.26 | 0.12 | 0.031 | −0.50 | −0.03 |
|  | CP equiv. | −2e-3 | 5e-3 | 0.648 | −0.01 | 0.01 |
|  | IMI equiv. | 0.01 | 0.02 | 0.610 | −0.03 | 0.05 |
|  | DZ equiv. | 0.12 | 0.18 | 0.522 | −0.26 | 0.49 |
|  |  |  |  |  |  |  |
| f. PANAS PA | Variables | Coefficient | Standard error | *P* | 95% Confidence interval | |
|  |  |  |  |  | Lower bound | Upper bound |
|  | Heart rate** | −0.36 | 0.12 | 0.004 | −0.59 | −0.12 |
|  | Sex (M=1, F=2) | 1.58 | 1.22 | 0.203 | −0.89 | 4.06 |
|  | Age | 0.11 | 0.09 | 0.215 | −0.07 | 0.28 |
|  | CP equiv. | 0.01 | 3e-3 | 0.157 | 0.00 | 0.01 |
|  | IMI equiv. | 0.01 | 0.01 | 0.556 | −0.02 | 0.03 |
|  | DZ equiv. | 0.13 | 0.14 | 0.343 | −0.14 | 0.41 |
|  |  |  |  |  |  |  |
| g. EQ-5D, VAS | Variables | Coefficient | Standard error | *P* | 95% Confidence interval | |
|  |  |  |  |  | Lower bound | Upper bound |
|  | Heart rate** | −0.98 | 0.34 | 0.007 | −1.67 | −0.29 |
|  | Sex (M=1, F=2) | 2.04 | 3.52 | 0.567 | −5.12 | 9.20 |
|  | Age | 0.08 | 0.25 | 0.751 | −0.42 | 0.58 |
|  | CP equiv. | −0.01 | 0.01 | 0.395 | −0.03 | 0.01 |
|  | IMI equiv. | −0.05 | 0.04 | 0.161 | −0.13 | 0.02 |
|  | DZ equiv. | −0.29 | 0.39 | 0.462 | −1.08 | 0.50 |
|  |  |  |  |  |  |  |
| h. EQ-5D, utility scores | Variables | Coefficient | Standard error | *P* | 95% Confidence interval | |
|  |  |  |  |  | Lower bound | Upper bound |
|  | Heart rate* | −0.01 | 0.00 | 0.016 | −0.02 | 0.00 |
|  | Sex (M=1, F=2) | −0.04 | 0.04 | 0.292 | −0.12 | 0.04 |
|  | Age | 5e-4 | 3e-3 | 0.851 | −5e-3 | 0.01 |
|  | CP equiv.* | −2e-4 | 1e-4 | 0.035 | −5e-4 | −2e-5 |
|  | IMI equiv.** | −1e-3 | 4e-4 | 0.006 | −2e-3 | −4e-4 |
|  | DZ equiv. | 4e-3 | 4e-3 | 0.303 | −0.01 | 4e-3 |
|  |  |  |  |  |  |  |
|  |  | | | | |  |

HR, heart rate; sdnn, standard deviation of the normal-to-normal interval; cvrr, coefficient of variation of R-R interval; STAI, State-Trait Anxiety Inventory; PANAS, the Positive and Negative Affect Schedule; PA, positive affect; NA, negative affect; EQ-5D-5L, EuroQol 5 dimensions 5-level; VAS, visual analog scale; CP equiv., chlorpromazine-equivalent; IMI equiv., imipramine-equivalent; DZ equiv., diazepam-equivalent.

**P*<0.05; ***P*<0.01.
