# Supplementary figures and images for: Feasibility of a wrist-worn wearable device for estimating mental health status in patients with mental illness
Source: Front Psychiatry. 2023 Jul 20;14:1189765. doi: 10.3389/fpsyt.2023.1189765 (PMC10399687; doi:10.3389/fpsyt.2023.1189765)

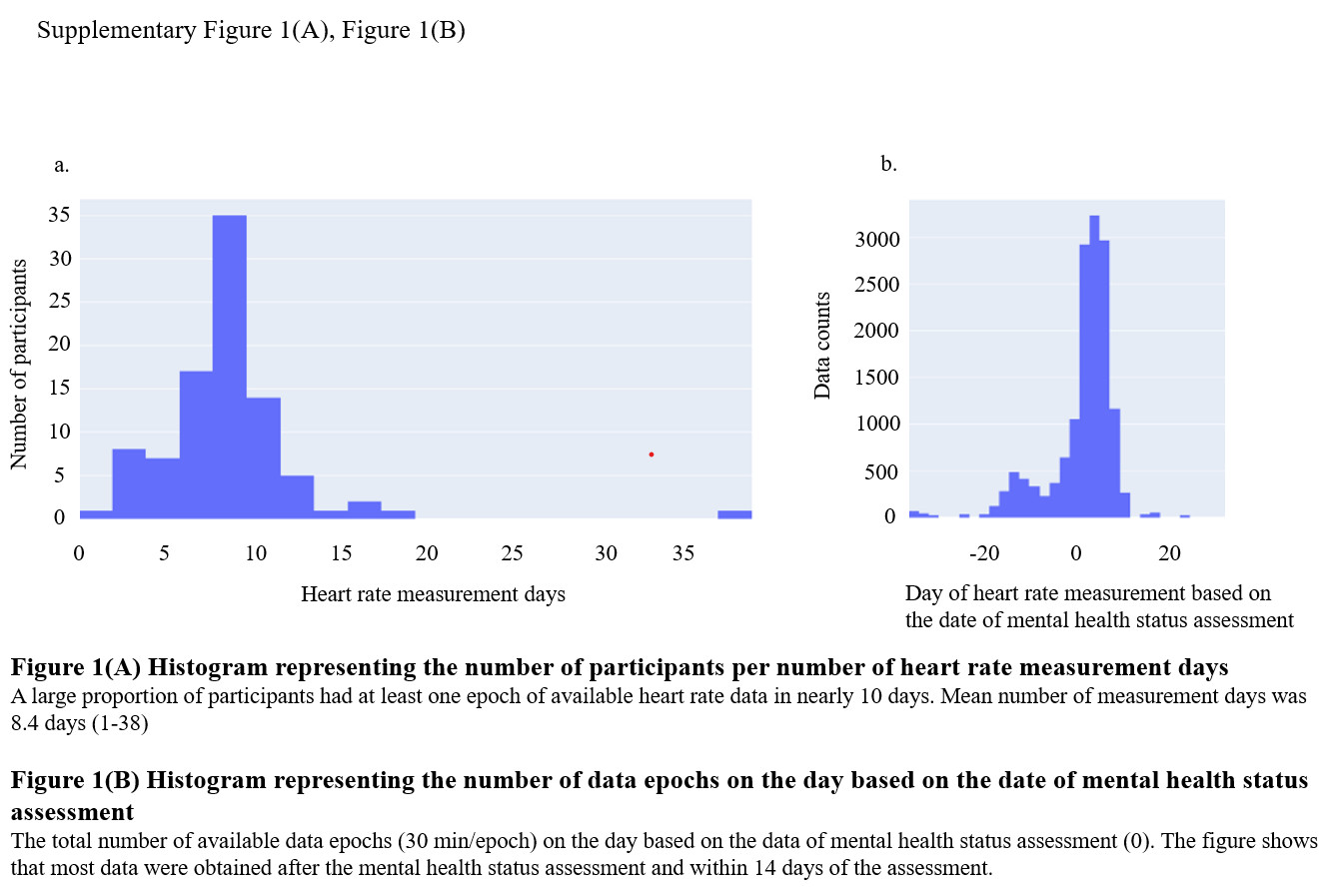

Supplement: Supplementary file 2 [file Image_1.JPEG]
